# Classification of induced malaria case in an elimination setting: investigation of transfusion-transmitted malaria cases

**DOI:** 10.1186/s12936-020-03203-x

**Published:** 2020-03-30

**Authors:** Mei-hua Zhang, Sui Xu, Ya-ping Gu, Yao-bao Liu, Hong Lin, Chao-yong Xie, Yue-e Chen, Jian-feng Chen, Hua-yun Zhou, Leonard Ortega, Guo-ding Zhu, Jun Cao

**Affiliations:** 1 National Health Commission Key Laboratory of Parasitic Disease Control and Prevention, Jiangsu Provincial Key Laboratory on Parasite and Vector Control Technology, Jiangsu Institute of Parasitic Diseases, Wuxi, 214064 Jiangsu People’s Republic of China; 2 Jiangsu Province Blood Center, Nanjing, 210042 Jiangsu People’s Republic of China; 3 Nanjing Municipal Center for Diseases Control and Prevention, Nanjing, 210003 Jiangsu People’s Republic of China; 4 Xuzhou Municipal Center for Diseases Control and Prevention, Xuzhou, 221002 Jiangsu People’s Republic of China; 5 Taizhou Municipal Center for Diseases Control and Prevention, Taizhou, 225300 Jiangsu People’s Republic of China; 6 World Health Organization, Geneva, Switzerland; 7 Center for Global Health, School of Public Health, Nanjing Medical University, Nanjing, 211166 Jiangsu People’s Republic of China; 8 Public Health Research Center, Jiangnan University, Wuxi, 214122 People’s Republic of China

**Keywords:** Malaria elimination, Indigenous malaria, Induced malaria, Transfusion-transmitted malaria

## Abstract

**Background:**

Since the National Malaria Elimination Action Plan was launched in China in 2010, local malaria transmission has decreased rapidly. Zero indigenous cases were reported since 2017. However, after 2010, the proportion of imported cases in China increased from 45.7% in 2010 to 99.9% in 2016, and almost all provinces of China have reported imported cases in recent years. Prevention of the reintroduction of malaria into China is crucial for the maintenance of its malaria-free status. Hence, it is of utmost importance to correctly identify the source of malaria infections within the country.

**Case introduction and response:**

In 2016 and 2017, three laboratory-confirmed cases of malaria caused by *Plasmodium falciparum* were identified in patients with no previous travel history to endemic areas were reported in Jiangsu Province, China, where malaria due to *P. falciparum* was eliminated about 30 years ago. These were diagnosed after 41, 31 and 39 days of seeking treatment, respectively, and all of them had received blood transfusions. Further investigations indicated that two of the cases had received blood from foreign students (from Indonesia and Ghana), and the other had received blood from an individual who had worked in Equatorial Guinea. All three blood donors were traced, and found to be carrying asymptomatic *P. falciparum* infections by microscopic examination and PCR. Furthermore, five polymorphic microsatellite markers (C1M4, C4M62, C13M13, C14M17, and C13M63) were typed and used to link parasites from the donors with those of the transfusion-receiving patients.

**Conclusions:**

Three transfusion-transmitted malaria cases were identified in China, all of which were due to the transfusion of blood donated by individuals who had contracted malaria outside the country. These cases can provide a reference for those faced with similar challenges in malaria case identification and classification in other regions. In addition, a stricter screening policy including the use of appropriate detection methods for malaria parasites should be developed and adopted for blood donation in regions undergoing malaria elimination.

## Background

Despite being preventable and treatable, malaria continues to have a devastating impact on the health and livelihood of people around the world. According to the World Health Organization (WHO), about 228 million malaria cases and an estimated 405,000 deaths occurred worldwide in 2018 [1].

The WHO launched the *Global technical strategy for malaria 2016*–*2030* in 2015, aiming to reduce global malaria mortality rates and malaria case incidence by at least 90% by 2030, and to eliminate malaria for at least 35 countries [2]. WHO certification of malaria elimination in a particular country requires evidence that local transmission of human malaria parasites has ceased, resulting in zero incidence of indigenous cases for at least three consecutive years [3].

China has made remarkable achievements in malaria control over the past decades. In 2010 it launched the National Malaria Elimination Programme, with the goal of achieving malaria elimination by 2020 [4]. Local malaria transmission has been significantly reduced, leading to the proportion of imported cases increasing from 45.7% in 2010 to 99.9% in 2016, with nearly all provinces reporting imported cases in the past few years [5]. No indigenous case has been reported since 2017 [6, 7]. However, occasionally, few cases are reported in which the patient had never been to any malaria endemic areas, which necessitates a search for other modes of transmission.

According to WHO guidelines, an *introduced malaria* case refers to a case contracted locally, with strong epidemiological evidence linking it directly to a known imported case (first-generation local transmission). An *induced case* refers to a case for which the origin can be traced to blood transfusion or other form of parenteral inoculation of the parasite but not to transmission by a natural mosquito-borne inoculation [3]. A clear distinction between induced and indigenous (or introduced) malaria cases has become especially important so as to provide sufficient evidence to exclude the possibility of the re-establishment of malaria transmission. This study reports the identification of three transfusion-transmitted malaria cases via imported cases to China in 2016 and 2017, which can provide a reference for the investigation and analysis of other cases of a similar nature.

## Case descriptions

In Jiangsu Province, malaria caused by *P. falciparum* was eliminated in the 1990s, and no indigenous *Plasmodium vivax* malaria cases have been reported since 2011 [8]. In 2016 and 2017, however, three cases of *P. falciparum* were reported from three different laboratories. None of the three patients had a history of travel to a malaria endemic country, and none had a past history of infection.

### Case A

A 64-year-old male admitted to Taizhou Traditional Chinese Medicine Hospital on 10th November 2016, was confirmed positive for *P. falciparum* infection on the 21st of December. The patient had been receiving treatment for hypertension for more than 10 years, and had undergone blood dialysis treatment since 2002 in this hospital. He had undergone 11 blood transfusions (supplied by Taizhou Blood Station) from 23 donors from 14 November to 21 December 2016.

### Case B

A 43-year-old female was admitted to Nanjing No.2 Hospital on 22 March 2017, and transferred to Nanjing Gulou Hospital, Jiangsu, on the 20th April where *P. falciparum* malaria was confirmed 2 days later. She had been receiving treatment for gynecological diseases. She had received 3 blood transfusions (supplied by Nanjing Blood Station and Jiangsu Province Blood Center) from 4 donors between the 22nd of March to the 22nd of April 2017.

### Case C

A 73-year-old female was admitted to Xuzhou Traditional Chinese Medicine Hospital on the 7th of May 2017, and a diagnosis of *P. falciparum* malaria was made on the 16th of June. She had been receiving treatment for myelodysplastic syndrome for about 4 years. During this treatment, she received 10 blood transfusions (supplied by Xuzhou Blood Station) from 10 donors between the 7th of May and the 16th of June 2017.

Although these cases were treated for different diseases, all share a history of blood transfusion, with onset of fever-related symptoms 9–13 days post-transfusion. In all cases, laboratory confirmation of the presence of *P. falciparum* infection occurred 2 or 3 weeks after blood transfusion (Table 1).

**Table 1:** The information of 3 transfusion-transmitted malaria cases and their donors

Cases	Recipient					Donor				
	Age	Sex	Date of blood transfusion	Date of onset	Date of diagnosis	Age	Sex	Occupation	Date of arriving China	Date of blood donation
A	64	M	24 Nov 2016	3 Dec 2016	21 Dec 2016	20	F	Foreign student	16 Nov 2016	19 Nov 2016
B	43	F	30 Mar 2017	12 Apr 2017	22 Apr 2017	22	M	Foreign student	1 Sep 2016	17 Mar 2017
C	73	F	13 May 2017	23 May 2017	16 Jun 2017	38	M	Chinese labourer	4 Apr 2017	5 May 2017

Further investigation was performed by Jiangsu Institute of Parasitic Diseases (JIPD) and local Centers for Disease Control and Prevention (CDCs), along with blood centres and the hospitals where patients received medical treatment, in Taizhou (Case A), Nanjing (Case B) and Xuzhou (Case C). To identify the origin of the infection, JIPD along with CDCs from Taizhou, Nanjing and Xuzhou carried out on-site investigations at the hospital and blood stations. All blood samples were tracked with reference to the blood transfusion records saved in the blood stations, and screened for malaria parasites by PCR. Blood donated by two foreign students (Donor of Case A from Indonesia, and Donor of Case B from Ghana) and one Chinese labourer who came back from Equatorial Guinea were found to be positive for malaria parasites (Table 2).

**Table 2:** Parasites detection and genotyping from donors and recipients

		Parasites detection			Microsatellite markers^a^				
		RDT	Microscopy	PCR	C1M4	C4M62	C13M13	C14M17	C13M63
Case A	Donor-stored	–	NA	+	117.27	248.42	163.93	195.11	153.58
	Donor-fresh	–	+	+	117.00	248.42	164.17	195.11	152.48
	Recipient	+	+	+	117.02	248.33	164.10	193.17	152.4
Case B	Donor-stored	–	NA	+	NA	214.68	151.88	164.84	NA
	Donor-fresh	–	+	+	NA	214.72	151.79	164.92	NA
	Recipient	+	+	+	NA	214.64	151.82	164.93	NA
Case C	Donor-stored	+	NA	+	NA	NA	NA	NA	NA
	Donor-fresh	+	+	+	NA	217.42	164.22	170.13	175.49
	Recipient	+	+	+	NA	217.02	163.96	169.92	175.43

*NA* not applicable

^a^The numbers indicate the allele sizes (in bp) corresponding to the major peak of each microsatellite

Face to face investigations of the three donors were conducted in order to ascertain their previous history of malaria infection, bed net usage behaviour and experience of blood donation. In addition, during the investigation, venous blood samples were collected from the three donors for rapid diagnostic tests (RDT, Guangzhou Wondfo Biotech Co., Ltd), microscopic examination and molecular-based analysis. *Plasmodium falciparum* malaria parasites were found via microscopic examination from all three donors, the parasitaemia of Case A, B and C were 2, 0.5, 120 parasites/μL respectively, and the presence of the parasite was confirmed by PCR in all three cases [9]. The RDT test was negative for both Case A and B donors and was positive for the Case C donor (Table 2).

To further investigate whether the parasites infecting the donors matched those found in the patients, five polymorphic microsatellite markers (C1M4, C4M62, C13M13, C14M17, and C13M63) were selected and amplified from the blood donors and recipients [10]. Genomic DNA was extracted using QIAamp DNA blood kit (Qiagen, Valencia, CA), as per the manufacturer’s protocol. Polymorphic microsatellite regions were identified in donor and recipient samples by fluorescent-labelled PCR typing. For Case A, all the five microsatellite markers were amplified successfully and were completetly identical between the donor and the patient. For Case B, three microsatellite markers (C4M62, C13M13, C14M17) were successfully amplified and were found to match between the donor and the recipient. For Case C, four microsatellite markers (C4M62, C13M13, C14M17, and C13M63) were amplified successfully and parasites from the donor and those from the recipient were found to match (Table 2).

## Discussion

Certification of malaria elimination in a country requires proof that local transmission of all human malaria parasites has been interrupted, resulting in zero incidence of indigenous cases for at least the past three consecutive years [3]. Evidence-based classification of all malaria cases is important for verification of countries and/or regions within countries approaching malaria elimination or maintaining malaria free status. However, classification of cases as imported, introduced, locally acquired, relapsing, recrudescent or induced is often difficult [3]. Sufficient information should be collected for identification of induced cases. For example, the three blood transfusion malaria cases described in this study, included epidemiological and parasitological data from the donors and the recipients, in addition to the relationships of molecular markers used for genotyping parasites from donors and recipients.

Three transfusion-transmitted induced malaria cases via imported cases to China have been reported, and an evidence-based approach is provided for investigation and analysis of other cases of a similar nature. Usually, a combination of epidemiological and parasitological evidence is enough to prove blood-transfusion transmission, however, further evidence of a link between donor and recipient provided by the molecular typing of parasite microsatellite markers as used in this study adds a further layer of certainty regarding the provenance of infection.

In regions entering malaria elimination phases, clinicians and laboratory staff may not consider malaria as a cause of febrile illness. All the three cases in this study were considered and confirmed as malaria at least 2 weeks subsequent to the onset of symptoms. This length of delay in diagnosis may lead to serious consequences for the patient, such as severe malaria and death [11]. In addition, if the patient recovers without treatment for malaria, they may continue to harbour the parasite and may act as a reservoir for further infections in regions that are still receptive to malaria transmission [12].

Furthermore, if malaria is not identified in the patient, then the donor would also remain undiagnosed and could also be a source of infection to malaria vectors leading to the re-introduction of malaria transmission. It is, therefore, necessary for clinicians and laboratory staff to maintain a high level of awareness and expertise regarding malaria case detection and treatment.

Due to social and economic development and the “Belt and Road Initiative”, the population has rapidly become more mobile, leading to increased threats for the maintenance of the malaria-free status of China [13, 14]. In this study, RDT tests were negative for malaria from two foreign students, which may be due to the low parasitaemia in the asymptomatic donors. Other countries where malaria incidence is very low may encounter similar situations in which malaria cases may not be identified by RDT tests or microscopical examination. Therefore, more stringent screening policies should be considered during blood donation in such settings [15]. Standard screening with more sensitive detection technologies such as PCR should be developed for the screening of blood from at-risk donors such as overseas students and migrant workers. In addition, the potential treatment of whole blood with parasite killing processes, such as UV light and riboflavin before storage in blood banks should be considered [16].

## Conclusion

Three transfusion-transmitted malaria cases were identified in China in 2016 and 2017. All cases were due to the donation of malaria parasite infected blood by individuals who had been infected outside China. These cases highlight a threat to the maintenance of China’s malaria-free status, and can provide a reference for those faced with similar challenges in malaria case identification and classification. In addition, a stricter screening policy including the use of appropriate detection methods for malaria parasites should be developed and adopted during blood donation in malaria elimination settings.

## Data Availability

All relevant data are included in this report.

## Abbreviations

WHO: World Health Organization
JIPD: Jiangsu Institute of Parasitic Diseases
PCR: Polymerase chain reaction
RDT: Rapid diagnostic tests
